# Effect of proton pump inhibitors on the risk of chronic kidney disease: A propensity score-based overlap weight analysis using the United Kingdom Biobank

**DOI:** 10.3389/fphar.2022.949699

**Published:** 2022-11-10

**Authors:** Xing-Yu Zhang, Qiang-Sheng He, Zhong Jing, Juan-Xia He, Jin-Qiu Yuan, Xiao-Yu Dai

**Affiliations:** ^1^ Department of Nephrology, Mianyang Central Hospital, School of Medicine, University of Electronic Science and Technology of China, Mianyang, China; ^2^ Clinical Research Center, Guangdong; Scientific Research Center, The Seventh Affiliated Hospital, Sun Yat-sen University, Shenzhen, China; ^3^ Department of Nephrology, Mianyang 404 Hospital, Mianyang, China; ^4^ Department of Information Engineering, University of Lanzhou City, Lanzhou, China

**Keywords:** proton pump inhibitors, chronic kidney disease, United Kingdom Biobank, cohort, propensity score weighting *via* overlap weights

## Abstract

**Background:** Proton pump inhibitors (PPIs) are widely used and have been linked to kidney diseases. However, the role of PPI use in the development of chronic kidney disease (CKD) remains unclear. We undertook this study to examine the association between PPI use and the subsequent risk of CKD.

**Methods:** This is a prospective analysis of 462,421 participants free of cancer diagnosis or chronic kidney disease from the United Kingdom Biobank. Self-reported PPI use was recorded using an electronic questionnaire and confirmed by a trained staff. Incident CKD was identified based on the medical history. Overlap propensity score weighting with the Cox model was used to calculate the effect of PPI use on CKD risk. The number needed to harm (NNH) was calculated at 5 and 10 years of follow-up.

**Results:** We documented 7,031 cases of CKD over a median follow-up of 8.1 years. Overlap propensity score weighting analysis showed that regular PPI users had a 37% higher risk of CKD incident than non-users (HR 1.37, 95% CI 1.28–1.47). The association persisted across subgroup analyses, different types of PPIs, and several sensitivity analyses. Quantitative bias analysis indicated that the result was robust to unmeasured confounding (E-value 2.08, lower 95% CI 1.88). The NNH was 147.9 and 78.6 for 5 and 10 years of follow-up, respectively. A head-to-head comparison showed that PPI users had a 19% higher risk of CKD than H2RA users (HR 1.19, 95% CI 1.02–1.39).

**Conclusion:** The regular use of PPI is associated with a higher risk of CKD. Healthcare providers should carefully weigh up the potential benefits against the risk in prescribing PPIs, particularly for patients requiring long-term treatment.

## Introduction

Proton pump inhibitors (PPIs) are primarily indicated for both the treatment and prevention of acid-related disorders, such as gastroesophageal reflux disease (GERD), peptic ulcers, and non-steroidal anti-inflammatory drug (NSAID)-related bleeding prophylaxis (Freedberg et al., 2017). Given the combination of high efficacy with low toxicity and safety of short-term use, PPIs are widely prescribed and are among the top 10 most frequently used drugs worldwide (Lindsley, 2014). However, the overutilization of PPIs became a health concern, and 40%–60% of people were taking PPIs with no appropriate indication (Gomm et al., 2016). Overutilization of PPIs not only increased healthcare expenditure but have also been linked to various adverse effects, such as *Clostridium difficile* infection, stroke, fractures, and diabetes (Schoenfeld and Grady, 2016; Freedberg et al., 2017; He et al., 2020; Xia et al., 2021; Yang et al., 2021; Yuan et al., 2021). Concerns have been raised about the increased risk of kidney disease among PPI users.

Chronic kidney disease (CKD) is the major cause of the early morbidity and mortality worldwide with a global prevalence of 9.1% in 2017, resulting in 35.8 million disability-adjusted life years and 1.2 million deaths (GBD Chronic Kidney Disease Collaboration, 2020). Risk factors influencing CKD are complex, including the unhealthy lifestyles, obesity, cardiovascular disease, diabetes mellitus, hypertension, and inappropriate drug use (Tuttle et al., 2019; Kalantar-Zadeh et al., 2021). The growing drug use, like NSAIDs, may also contribute to the higher prevalence of CKD (Hsu et al., 2015; Tuttle et al., 2019). There is accumulating evidence that PPI use might affect kidney function and thus result in CKD (Xie et al., 2016; Al-Aly et al., 2020; Vengrus et al., 2021). For example, a meta-analysis of six cohorts and two case–control studies found that PPI use was associated with a 35% higher risk of CKD [risk ratios (RR) 1.35, 95% confidence interval (CI), 1.15–1.56] (Vengrus et al., 2021). These observational studies are thought-provoking but have important limitations to the evidence base, such as either inaccurate exposure measurements through retrospective recall or administrative claims data or nonavailability of potential confounders such as lifestyle factors (Freedberg et al., 2017). A recent randomized controlled trial (RCT) consisting of 17,598 patients with cardiovascular disease showed that pantoprazole seemed to have a 17% non-significantly higher risk of CKD than a placebo (OR 1.17; 95% CI 0.94–1.15) (Moayyedi et al., 2019). However, this trial was questioned with a short period of follow-up and relatively small sample size (Losurdo et al., 2020; Simin et al., 2020).

Given the extensive use of PPIs and the public health threat of CKD, understanding the role of PPI use in the development of CKD has important implications for clinical practice and public health. Therefore, we performed a prospective cohort study to examine the association between PPI use and incident CKD in the general population using the United Kingdom Biobank dataset.

## Methods

### Study and participants

The United Kingdom Biobank is a well-designed ongoing prospective cohort study of over 500,000 individuals aged 37–73 years who were recruited from 21 assessment centers across the United Kingdom. In 2006–2010, eligible participants were invited to visit the closest assessment center to complete the baseline assessment, including a touchscreen questionnaire, physical measures, and biological sample collection. Further details about the United Kingdom Biobank cohort have been published elsewhere (Sudlow et al., 2015). The United Kingdom Biobank study has been approved by the North West Multi-Center Research Ethics Committee, the England and Wales Patient Information Advisory Group, and the Scottish Community Health Index Advisory Group. All participants provided written informed consent prior to data collection. In this study, we excluded 36,856 participants with prior cancer diagnosis, 1,953 participants who had chronic kidney disease diagnosis before baseline, and 1,298 who subsequently withdrew from the study. In total, our analysis included 462,421 participants.

### Assessment of proton pump inhibitor use

At baseline, regular use of PPI was first recorded using an electronic questionnaire and then confirmed by a United Kingdom Biobank nurse (Yang et al., 2021; Yang et al., 2021; Xia et al., 2021). The recorded type of PPIs included omeprazole, lansoprazole, pantoprazole, rabeprazole, and esomeprazole. “Regular use” for medications in the United Kingdom Biobank was defined as taking PPIs in most days of the week for the last 4 weeks.

### Assessment of the outcome

Incident CKD was identified based on medical history and linkage to data on primary care and hospital admissions. We used the chronic kidney disease variables provided by the United Kingdom Biobank, which integrated information from these different data sources (including primary care, hospital admissions, self-report, and death register). Details of the algorithms used to identify CKD could be found on the United Kingdom Biobank website (www.ukbiobank.ac.uk).

### Covariates

Covariate information was obtained from a touchscreen questionnaire and verbal interview at baseline. Sociodemographic factors (age, sex, and ethnicity) and lifestyle factors (smoking status, alcohol consumption, and dietary intake) were self-reported. The index of multiple deprivations based on the postcode of residence was determined as a composite measure of the socioeconomic status. Blood pressure was measured by the research staff. Current concomitant comorbidities (hyperlipidemia, diabetes, cardiovascular disease, GERD, and peptic ulcer) were assessed using the same way to assess CKD. Other medication use, including aspirin, non-aspirin NSAIDs, acetaminophen, antihypertensive drugs, statin, metformin, and histamine-2 receptor antagonists (H2RAs), were assessed using the same way to assess PPI use.

### Statistical analysis

We calculated person-year of follow-up from the recruitment date to the date of first diagnosis of CKD, death, or the last date of follow-up (31 October 2017 for England and Wales and 31 October 2016 for Scotland), whichever came first. We fitted Cox proportional hazards models with age as the timescale to estimate the effect of PPI use on CKD. Given that PPI users are more likely to have underlying comorbidities, overlap propensity score weighting was used to address potential confounding. This newly developed method of overlap weighting avoids excluding study participants from the available sample and eliminates the potential for outlier weights. A propensity score for taking PPIs was calculated by multivariate logistic regression model conditional on baseline covariates, including age, sex, ethnicity, socioeconomic status, smoking status, alcohol consumption, physical activity, fruit and vegetable intake, BMI, current medical status (*systolic blood pressure*, *hyperlipidemia*, *diabetes*, *cardiovascular disease*, *GERD*, *and peptic ulcer*), and concomitant medication use (*including aspirin*, *non-aspirin NSAIDs*, *acetaminophen*, *antihypertensive drugs*, *statin*, *metformin*, *and H2RAs*). The overlap weighting method was then applied, in which each individual’s weight is the probability of that individual belonging to the opposite treatment group (Thomas et al., 2020). We assessed the balance of all covariates between the two groups using standardized mean differences (SMDs), with SMD less than 0.1 considered negligible. Weighted Kaplan–Meier curves and weighted Cox models were fitted to calculate marginal HRs with 95% CIs of PPI use on CKD risk. Proportional hazard assumption was checked using Schoenfeld’s tests, and no violation was detected. Missing data were treated as a separate category for other covariates. For easy interpretation, the number needed to harm (NNH) was calculated according to Altman and Andersen (1999). Since H2RAs are used for similar indications as PPIs, we investigated the association between regular H2RA use and CKD risk and conducted a head-to-head comparison between PPIs and H2RAs.

We conducted additional stratified analyses to evaluate potential effect modifications of sex, age, obesity, smoking, drinking status, hypertension, diabetes, regular use of aspirin and NSAIDs, GERD, and clinical indication for PPI use. To test the robustness of our findings, we conducted several sensitivity analyses. First, we lagged the exposure for 2 years to minimize the potential for reverse causation. Second, we excluded participants with cardiovascular disease to minimize the potential impact of health status. Third, as alternatives to the overlap-weighting method used in our primary analysis, we used the stabilized inverse probability treatment-weighting and propensity score-matching analysis methods to control for the influence of confounding variables. Fourth, we fitted the multivariable-adjusted Cox model to control for confounding variables. Last, to evaluate the potential influence of unmeasured confounders, we calculated the E-value, which quantifies the minimum strengths of association between an unmeasured confounder and exposure or outcome, conditional on measured covariates that would be necessary to fully explain away the observed association between PPI use and CKD risk (VanderWeele and Ding, 2017). All analyses were conducted using R software (version 3.5.0, R Foundation for Statistical Computing, Vienna, Austria).

## Results

This study included 462,421 participants in the final analyses, of which 45,046 (9.74%) participants reported regular use of PPIs. PPI users tended to be deprived, consumed less alcohol, less physically active, with a higher rate of comorbidities (diabetes, hyperlipidemia, and CVD), and were more likely to take other medications (like aspirin, antihypertensive drugs, NSAIDs, statin, metformin, and H2RAs) (Table 1). As expected, PPI users had a higher rate of upper gastrointestinal bleeding, GERD, and peptic ulcer.

**TABLE 1:** Baseline characteristics of participants by PPI use before and after weighting.

	Before weighting			After weighting^a^		
	Non-regular PPI user	Regular PPI user	SMD	Non-regular PPI user	Regular PPI user	SMD
N	417375	45046		28150.78	28150.78	
Mean (SD) age, years	55.93 (8.12)	59.36 (7.32)	0.443	58.89 (7.48)	58.89 (7.49)	<0.001
Male	193729 (46.4)	20540 (45.6)	0.016	12742.1 (45.3)	12742.1 (45.3)	<0.001
White people	394038 (94.4)	42817 (95.1)	0.029	26675.3 (94.8)	26675.3 (94.8)	<0.001
Index of multiple deprivation quintile			0.185			<0.001
1 (least deprived)	83218 (19.9)	6968 (15.5)		4552.9 (16.2)	4552.9 (16.2)	
2	82078 (19.7)	8054 (17.9)		5,137.5 (18.2)	5,137.5 (18.2)	
3	81879 (19.6)	8387 (18.6)		5,284.5 (18.8)	5,284.5 (18.8)	
4	80918 (19.4)	9087 (20.2)		5,622.5 (20.0)	5,622.5 (20.0)	
5 (most deprived)	78626 (18.8)	11481 (25.5)		6868.0 (24.4)	6868.0 (24.4)	
Missing	10656 (2.6)	1,069 (2.4)		685.3 (2.4)	685.3 (2.4)	
Smoking status			0.184			<0.001
Current	43749 (10.5)	5,101 (11.3)		3225.0 (11.5)	3225.0 (11.5)	
Previous	138798 (33.3)	18619 (41.3)		11235.9 (39.9)	11235.9 (39.9)	
Never	234828 (56.3)	21326 (47.3)		13689.9 (48.6)	13689.9 (48.6)	
Alcohol consumption			0.226			<0.001
Daily or almost daily	85788 (20.6)	7974 (17.7)		5,137.0 (18.2)	5,137.0 (18.2)	
One to four times a week	208415 (49.9)	19470 (43.2)		12392.5 (44.0)	12392.5 (44.0)	
One to three times a month	46234 (11.1)	5,170 (11.5)		3238.0 (11.5)	3238.0 (11.5)	
Special occasions only/never	76938 (18.4)	12432 (27.6)		7383.3 (26.2)	7383.3 (26.2)	
Physical activity			0.191			<0.001
Low	61445 (14.7)	8322 (18.5)		4998.6 (17.8)	4998.6 (17.8)	
Moderate	137428 (32.9)	13465 (29.9)		8566.7 (30.4)	8566.7 (30.4)	
High	138102 (33.1)	12202 (27.1)		7887.9 (28.0)	7887.9 (28.0)	
Unknown/missing	80400 (19.3)	11057 (24.5)		6697.5 (23.8)	6697.5 (23.8)	
Fruit and vegetable intake ≥5 portions per day	156661 (37.5)	17009 (37.8)	0.005	10726.4 (38.1)	10726.4 (38.1)	<0.001
Mean (SD) body mass index	27.22 (4.69)	29.23 (5.12)	0.409	28.91 (5.41)	28.91 (5.04)	<0.001
Mean (SD) systolic blood pressure	139.40 (19.66)	141.86 (19.09)	0.127	142.25 (19.68)	141.37 (19.09)	0.045
Diabetes	22541 (5.4)	5,203 (11.6)	0.222	3014.5 (10.7)	3014.5 (10.7)	<0.001
Hyperlipidemia	208459 (49.9)	31283 (69.4)	0.406	18638.9 (66.2)	18638.9 (66.2)	<0.001
Cardiovascular disease	23381 (5.6)	8503 (18.9)	0.414	4549.2 (16.2)	4549.2 (16.2)	<0.001
Gastroesophageal reflux disease	8730 (2.1)	18201 (40.4)	1.06	5,380.1 (19.1)	5,380.1 (19.1)	<0.001
Peptic ulcer	4249 (1.0)	5,545 (12.3)	0.465	1963.0 (7.0)	1963.0 (7.0)	<0.001
Upper gastrointestinal bleeding	2155 (0.5)	1,335 (3.0)	0.188	592.1 (2.1)	592.1 (2.1)	<0.001
Aspirin use	54297 (13.0)	11037 (24.5)	0.298	6502.4 (23.1)	6502.4 (23.1)	<0.001
Paracetamol use	87693 (21.0)	14773 (32.8)	0.268	8731.0 (31.0)	8731.0 (31.0)	<0.001
NASID use	67736 (16.2)	7767 (17.2)	0.027	5,149.7 (18.3)	5,149.7 (18.3)	<0.001
Antihypertensive drugs	76523 (18.3)	16722 (37.1)	0.429	9563.6 (34.0)	9563.6 (34.0)	<0.001
Statin use	59009 (14.1)	14728 (32.7)	0.449	8256.4 (29.3)	8256.4 (29.3)	<0.001
Metformin use	10497 (2.5)	2628 (5.8)	0.167	1,511.4 (5.4)	1,511.4 (5.4)	<0.001
H2RA use	7460 (1.8)	2028 (4.5)	0.156	1,382.5 (4.9)	1,382.5 (4.9)	<0.001

Values are numbers (percentages) unless otherwise stated.

^a^ Pseudopopulation created by applying the overlap propensity score weighting approach.

H2RAs, histamine-2 receptor antagonists; NSAIDs, non-steroidal anti-inflammatory drugs; PPI, proton pump inhibitor; SMD, standardized mean difference.

**FIGURE 1:**
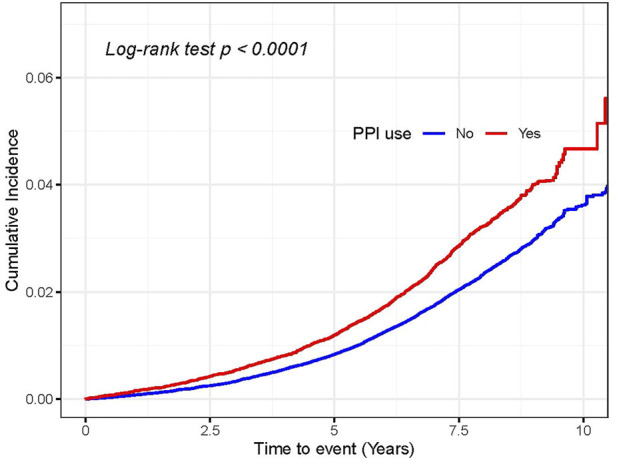
Cumulative incidence of chronic kidney disease in the overlap propensity score-weighted populations. The overlap weight-adjusted Kaplan–Meier curves were generated based on the propensity score. Also, the propensity score was derived using multivariate logistic regression conditional on the aforementioned covariates in Table 2. The log-rank test was used to compare the survival difference between PPI users and non-users.

During a median follow-up of 8.1 years, we documented 1,582 incident CKD events among the 45,046 PPI users (4.45 per 1,000 person-years) and 5,449 events among 417,375 non-users (1.63 per 1,000 person-years). In the crude model, regular PPI users had 2.05 times higher risk of incident CKD than non-users (HR 2.05, 95% CI 1.94–2.17) (Table 2). The overlap propensity score-weighted HR for CKD was attenuated to some extent, but remained significant (HR 1.37, 95% CI 1.28–1.47). The weighted cumulative incidence curve showed a similar result (Figure 1). For easy interpretation, NNHs were calculated based on the weighted HR and the CKD incidence among non-PPI users. Every 677.9 (95% CI, 646.5–737.0), 147.9 (95% CI, 137.3–166.2), and 78.6 (95% CI, 71.9–89.9) PPI users may result in one CKD case over 1, 5, and 10 years, respectively (Supplementary Figure S1).

**TABLE 2:** Association between regular use of proton pump inhibitors and the risk of chronic kidney disease.

	Cases/person-years	Incidence rate/	Hazard ratio [95% confidence interval]	
		1,000 person-years	Crude model	Propensity score-weighted model^a^
Non-regular PPI user	5,449/3350774	1.63	1.00 (references)	1.00 (references)
Regular PPI user	1,582/355293	4.45	2.05 (1.94, 2.17)	1.37 (1.28, 1.47)

^a^ Overlap weighted Cox model. The propensity score was derived using multivariate logistic regression conditional on age, sex (male, female), ethnicity (white people or others), socioeconomic status (the index of multiple deprivation, fifth), smoking status (never smoker, previous smoker, or current smoker), alcohol consumption (daily or almost daily, one to four times a week, one to three times a month, and special occasions only or never), physical activity (low, moderate, or high), fruit and vegetable intake (≥5 portions or <5 portions), body mass index, systolic blood pressure, concomitant comorbidities (hyperlipidemia, diabetes, cardiovascular disease, gastroesophageal reflux disease, and peptic ulcer, yes or no), and medication use (including aspirin, non-aspirin NSAIDs, acetaminophen, antihypertensive drugs, statin, metformin, and H2RAs). PPI, proton pump inhibitor; H2RAs, histamine-2 receptor antagonists; NSAIDs, non-steroidal anti-inflammatory drugs.

We also observed a similar association between PPI use and acute kidney injury (AKI). In the overlap weighted Cox model, regular PPI users had a 41% greater risk of AKI (HR 1.41, 95% CI 1.32–1.51) (Supplementary Table S1). Given that H2RAs are used for similar indications as PPIs, we also investigated the association between the regular use of H2RAs and CKD risk. In the crude model, the regular use of H2RAs was associated with a 65% greater risk of CKD (HR 1.65, 95% CI 1.45–1.86). However, this association disappeared in the overlap weighted Cox model (HR 1.10, 95% CI 0.96–1.25) (Supplementary Table S2). A head-to-head comparison showed that PPI users had a 19% greater risk of CKD than H2RA users (HR 1.19, 95% CI 1.02–1.39) (Supplementary Table S3).

**FIGURE 2:**
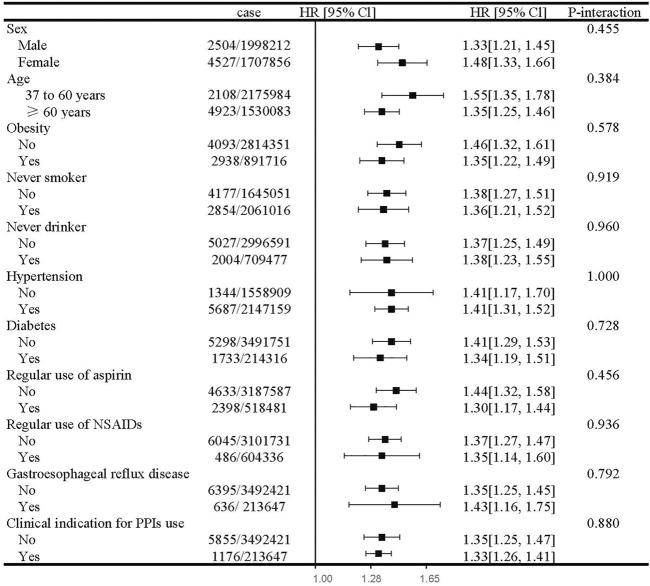
Subgroup analyses of regular use of proton pump inhibitors and risk of chronic kidney disease. Abbreviation: NSAIDs, non-steroidal anti-inflammatory drugs; PPI, proton pump inhibitor; HR, hazard ratio; CI, confidence interval. Estimated effects were based on the fully adjusted model (see the footnote in Table 2).

In subgroup analyses, the estimated risk of CKD with PPI use did not differ by sex, age, obesity, smoking, drinking status, hypertension, diabetes, regular use of aspirin and NSAIDs, GERD, and clinical indication for PPI use (Figure 2). For the individual class of PPIs, we observed a similar positive association with the risk of CKD (Table 3).

**TABLE 3:** Risk of chronic kidney disease by the class of proton pump inhibitors.

	Cases/person-years	Hazard ratio [95% confidence interval]	
		Crude model	Propensity score-weighted model^a^
Type of PPIs			
No PPI use	5,449/3350774	1.00 (References)	1.00 (References)
Omeprazole	966/230206	1.94 (1.81, 2.08)	1.35 (1.24, 1.47)
Lansoprazole	609/123880	1.89 (1.16, 1.23)	1.40 (1.26, 1.54)
Other PPI types	142/33184	1.89 (1.60, 1.23)	1.34 (1.11, 1.62)

^a^ Estimated effects were based on the propensity score-weighted model (see the footnote in Table 2).

Abbreviation: PPI, proton pump inhibitor.

Our primary results remained unchanged in several sensitivity analyses. The primary result did not change after lagging the exposure for 2 years (HR 1.51, 95% CI 1.40–1.63), excluding the participants with cardiovascular disease (HR 1.47, 95% CI 1.33–1.62), in the stabilized inverse probability of treatment weighting analysis (HR 1.43, 95% CIs 1.27–1.62), in the propensity score-matching analysis (HR 1.32, 95% CIs 1.21–1.43), and in the multivariable-adjusted analysis (HR 1.43, 95% CIs 1.34–1.53) (Supplementary Table S4). In the quantitative bias analysis, the E-value for the primary estimate was 2.08, and the lower 95% confidence limit for the E-value was 1.88 (Supplementary Figure S2). The E-value indicated that the observed risk ratio of 1.37 in PPI users could be fully explained by an unmeasured confounder that would be strongly associated with both PPI use and CKD incidence by a risk ratio of 2.08-fold each or above.

## Discussion

In this prospective population-based cohort study of over 0.46 million participants, we found that the regular use of PPI was associated with a 37% higher risk of CKD after adjustment for known confounders (weighted HR 1.37, 95% CI 1.28–1.47). The association between PPI use and CKD persists through subgroup analyses, different types of PPIs, and several sensitivity analyses. Additional analysis showed that, when directly compared with the use of H2RAs, which are less potent acid suppressors, the PPI use was associated with a 19% higher risk of CKD.

There is increasing evidence from observational studies suggesting that the long-term use of PPIs was associated with a higher risk of kidney disease, such as interstitial nephritis, AKI, and CKD (Xie et al., 2016; Qiu et al., 2018; Al-Aly et al., 2020). Lazarus et al. (2016) using the data on 10,482 participants in the Atherosclerosis Risk in Communities Study, found that PPI users had a 50% higher risk of CKD (HR 1.50, 95% CI 1.14–1.96) and validated these findings in a second large cohort of 248,751 patients. In addition, Arora et al. (2016) and Xie et al. (2016) also found that PPI use was associated with a 10–28% higher risk of CKD. Some meta-analysis studies summarized the aforementioned three studies and indicated that PPI use was associated with a higher risk of CKD (Wijarnpreecha et al., 2017; Qiu et al., 2018; Al-Aly et al., 2020; Vengrus et al., 2021). However, a lack of adjustment for several important confounders, such as diet, alcohol intake, and physical activity, might introduce bias in the findings (Desbuissons and Mercadal, 2019). In this study, we controlled for these confounders using the overlap weighting approach and confirmed that PPI use could increase the risk of CKD.

In addition to the aforementioned observational studies, a large RCT was conducted to evaluate the safety of pantoprazole among 17,598 participants with a median follow-up of 3 years. The researchers found that pantoprazole seemed to have a 17% non-significantly higher risk of CKD than the placebo (OR 1.17; 95% CI 0.94–1.15) (Moayyedi et al., 2019). However, this trial also had some limitations, such as insufficient power, a short follow-up period, potential selection bias, and possible conflicts of interest (Losurdo et al., 2020; Simin et al., 2020). Since sufficiently powered RCTs with adequate follow-up duration to evaluate the long-term safety of PPIs are unlikely to be conducted in the near future, the well-designed observational studies can be useful to investigate the long-term efficacy and safety of medications. Overlap weighting can mimic attributes of RCT and avoid some limitations of classic propensity score matching and weighting (Thomas et al., 2020). In our study, we found that PPI users had a higher BMI and higher rates of multiple comorbidities like GERD and diabetes. We used the overlap-weighting approach to adjust for underlying confounding factors, which led to precise balance on these critical variables at baseline. Overlap propensity score-weighted analysis showed that PPI users had a higher risk of CKD.

The underlying mechanism of the association between PPI use and CKD remains unclear. One possible explanation is that PPIs could increase the risk of acute interstitial nephritis (AIN) (Blank et al., 2014; Nochaiwong et al., 2018), which may develop to chronic interstitial nephritis and subsequently result in the development of CKD (Moledina and Perazella, 2016). A previous study revealed that a significant proportion of patients that suffered PPI-induced AIN did not recover to baseline, having either partial or no renal recovery, possibly because of rapid progression of AIN from inflammatory interstitial cellular infiltrates to interstitial fibrosis and chronic interstitial nephritis, especially in those patients with delayed diagnosis or treatment (Moledina and Perazella, 2016; Xie et al., 2016). Therefore, CKD might be a long-term complication and consequence of PPI-induced AIN due to the incomplete recovery of renal function and chronic interstitial nephritis (Moledina and Perazella, 2016). In addition, PPI use has been linked to hypomagnesemia, which could cause endothelial dysfunction by promoting atherosclerosis, inducing inflammation and inhibiting endothelial proliferation (Ferrè et al., 2010; Dousdampanis et al., 2014), which consequently results in the development of CKD (Dousdampanis et al., 2014). In addition, previous studies found that PPI use was associated with the gut microbiota alterations (Jackson et al., 2016) and diabetes (He et al., 2020; Yuan et al., 2021), which in turn may increase the risk of CKD (Dousdampanis et al., 2014; Yang et al., 2018). More research is required to investigate the underlying mechanisms.

The key strength of this study is that it lies in a well-established prospective cohort, which collected detailed information on lifestyle factors, medication use, and health conditions. We were able to fully investigate potential confounding factors that were often not available in administrative medical databases. In addition, the large sample size and event number enabled us to get the precisely estimated effects for the individual class of PPIs and subgroups. Lastly, a wide range of robust sensitivity analyses enhanced the validity of our findings.

This study had several limitations. First, owing to the observational nature of the study, we cannot fully exclude the potential confounding effect. PPI users might have poor health status and be more concerned about their personal health, so they may be more likely to receive CKD-related tests. Nonetheless, we used several steps to minimize potential confounding. We used the overlap-weighting approach to adjust for underlying confounding factors, which can mimic the attributes of a RCT and achieve balance of these critical variables at baseline. Furthermore, our primary result was robust to unmeasured confounding factors based on quantitative bias analysis, which indicated that an unmeasured confounder would need to be strongly associated with PPI use and CKD incidence to fully explain the observed association. We have controlled for major confounders in our primary analysis, suggesting that the observed association was unlikely caused by unmeasured confounders. Second, because information about PPI use was collected only once in baseline, we were unable to investigate the effects of time by varying the covariates and exposures on CKD. Last, we could not investigate the possible dose–response relationship due to insufficient information on dose and duration of PPI use.

## Conclusion

This large cohort study found that the regular use of PPIs was associated with a higher risk of CKD. Although the causal relationship cannot be determined with an observational study, given the large number of PPI users as well as the public health threat of CKD, healthcare providers should carefully weigh up the potential benefits against the risk in prescribing PPIs, particularly for patients requiring long-term continuous use. Further research studies are required to confirm our findings and to explore the underlying mechanisms.

## Data Availability

The data analyzed in this study is subject to the following licenses/restrictions: The study was conducted using the UKB resource (application number 51671, approved August 2019). The dataset can be obtained from the United Kingdom Biobank. Requests to access the dataset should be directed to https://www.ukbiobank.ac.uk.
