# Supplementary material for: Effect of proton pump inhibitors on the risk of chronic kidney disease: A propensity score-based overlap weight analysis using the United Kingdom Biobank
Source: Front Pharmacol. 2022 Nov 10;13:949699. doi: 10.3389/fphar.2022.949699 (PMC9685407; doi:10.3389/fphar.2022.949699)
Supplement: Supplementary file 1 [file Table1.DOCX]

| **Table S1**. Association between regular use of proton pump inhibitor and the risk of acute kidney injury | | | | |
| --- | --- | --- | --- | --- |
|  | Cases/ Person-years | Incidence rate /1000 person-years | Hazard Ratio [95% Confidence Interval] | |
|  |  |  | Crude model | Propensity score weighted- model^†^ |
| Non-regular PPI user | 5046/3349523 | 1.51 | 1.00[Reference] | 1.00[Reference] |
| Regular PPI user | 1782/ 354367 | 5.02 | 2.40[2.27, 2.53] | 1.41[1.32, 1.51] |

† Overlap weighted Cox model. Propensity score was derived by multivariate logistic regression conditional on age, sex (male, female), ethnicity (white, or other), socioeconomic status (index of multiple deprivation, fifth), smoking status (never smoker, previous smoker, or current smoker), alcohol consumption (daily or almost daily, one to four times a week, one to three times a month, special occasions only or never), physical activity (low, moderate, or high), fruit and vegetable intake (≥5 portions or <5 portions), body mass index, systolic blood pressure, concomitant comorbidities (hyperlipidemia, diabetes, cardiovascular disease, gastroesophageal reflux disease, peptic ulcer, yes or no), and medications use (including aspirin, non-aspirin NSAIDs, acetaminophen, antihypertensive drugs, statin, metformin, and H2RAs). H2RAs, histamine-2 receptor antagonists; NSAIDs, non-steroidal anti-inflammatory drugs.

| **Table S2**. Association between regular use of H2RAs and risk of chronic kidney disease | | | | |
| --- | --- | --- | --- | --- |
|  | Cases/ Person-years | Incidence rate /  1,000 person-years | Hazard Ratio [95% Confidence Interval] | |
|  |  |  | Crude model | Propensity score weighted-model^†^ |
| Non-regular H2RAs user | 6772/3630562 | 1.87 | 1.00[Reference] | 1.00[Reference] |
| Regular H2RAs user | 259/75505 | 3.43 | 1.65[1.45, 1.86] | 1.10[0.96, 1.25] |

† Overlap weighted Cox model. Propensity score was derived by multivariate logistic regression conditional on age, sex (male, female), ethnicity (white, or other), socioeconomic status (index of multiple deprivation, fifth), smoking status (never smoker, previous smoker, or current smoker), alcohol consumption (daily or almost daily, one to four times a week, one to three times a month, special occasions only or never), physical activity (low, moderate, or high), fruit and vegetable intake (≥5 portions or <5 portions), body mass index, systolic blood pressure, concomitant comorbidities (hyperlipidemia, diabetes, cardiovascular disease, gastroesophageal reflux disease, peptic ulcer, yes or no), and medications use (including aspirin, non-aspirin NSAIDs, acetaminophen, antihypertensive drugs, statin, metformin, and proton pump inhibitors). H2RAs, histamine-2 receptor antagonists; NSAIDs, non-steroidal anti-inflammatory drugs.

| Table S3. Head-to-head comparisons of PPIs versus H2RAs on risk of chronic kidney disease | | | | |
| --- | --- | --- | --- | --- |
|  | Cases/ Person-years | Incidence rate /1000 person-years | Hazard Ratio [95% Confidence Interval] | |
|  |  |  | Crude model | Propensity score weighted-model^†^ |
| Regular H2RA user | 180/59503 | 3.03 | 1.00[Reference] | 1.00[Reference] |
| Regular PPI user | 1503/339291 | 4.43 | 1.25[1.07, 1.46] | 1.19[1.02, 1.39] |

† Overlap weighted Cox model. Propensity score was derived by multivariate logistic regression conditional on age, sex (male, female), ethnicity (white, or other), socioeconomic status (index of multiple deprivation, fifth),smoking status (never smoker, previous smoker, or current smoker), alcohol consumption (daily or almost daily, one to four times a week, one to three times a month, special occasions only or never), physical activity (low, moderate, or high), fruit and vegetable intake (≥5 portions or <5 portions), body mass index, systolic blood pressure, concomitant comorbidities (hyperlipidemia, diabetes, cardiovascular disease, gastroesophageal reflux disease, peptic ulcer, yes or no), and medications use (including aspirin, non-aspirin NSAIDs, acetaminophen, antihypertensive drugs, statin, metformin). PPI, proton pump inhibitor; H2RAs, histamine-2 receptor antagonists; NSAIDs, non-steroidal anti-inflammatory drugs.

| Table S4. Sensitivity analyses of proton pump inhibitors and risk of chronic kidney disease | | |
| --- | --- | --- |
|  | Cases/ Person-years | HR [95%CI] |
| **Lagging the exposure for 2 years to allow a time window for CKD incidence ^*†^** | | |
| Non-regular PPI user | 5056/3348864 | 1.00[Reference] |
| Regular PPI user | 1435/354834 | 1.51[1.40, 1.63] |
| **Excluding the participants with cardiovascular disease at baseline ^*†^** | | |
| Non-regular PPI user | 4353/3168665 | 1.00[Reference] |
| Regular PPI user | 965/289929 | 1.47 [1.33, 1.62] |
| **Stabilized inverse probability of treatment weighting analysis ^†^** | | |
| Non-regular PPI user | 5449/ 3350774 | 1.00[Reference] |
| Regular PPI user | 1582/355293 | 1.43 [1.27, 1.62] |
| **Using propensity score–matching analysis^†^** | | |
| Non-regular PPI user | 930/275019 | 1.00[Reference] |
| Regular PPI user | 1194/274182 | 1.32 [1.21, 1.43] |
| **Multivariable-adjusted analysis^††^** | | |
| Non-regular PPI user | 5449/ 3350774 | 1.00[Reference] |
| Regular PPI user | 1582/355293 | 1.43 [1.34, 1.53] |

^*^: Estimated effects were based on the overlap weighted Cox model.
 ^†^: Propensity score was derived by multivariate logistic regression conditional on baseline covariates (see footnote in table 2).
**^††:^** Multivariable-adjusted Cox model was fitted with adjustment for baseline covariates (see footnote in table 2).

**
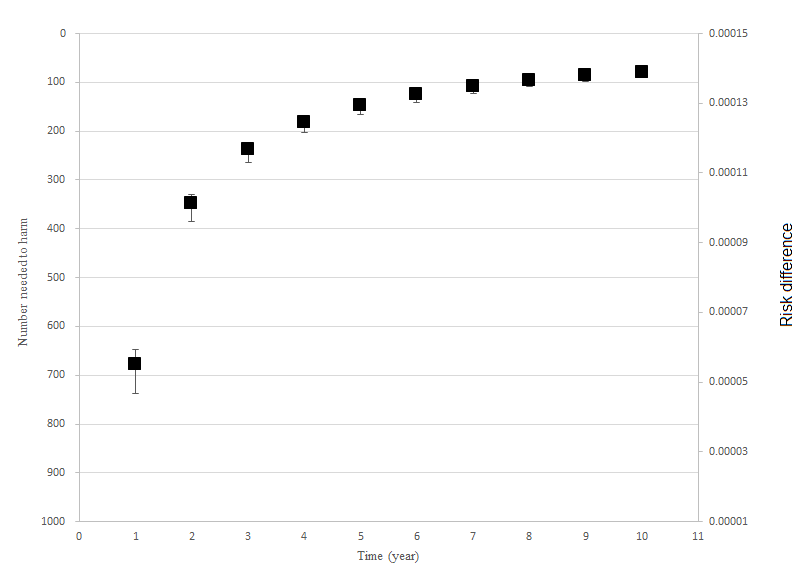
**

**Figure S1. Estimated number needed to harm for regular users of proton pump inhibitors use and risk of** **chronic kidney disease.**

The estimated number needed to harm was based on the fully adjusted HR of regular PPI use versus non-use (1.37, 95%CI 1.28 to 1.47) and diabetes rate in the non-user group (1.63 cases/1000 person-years), with the method described by Altman *et al*.

**
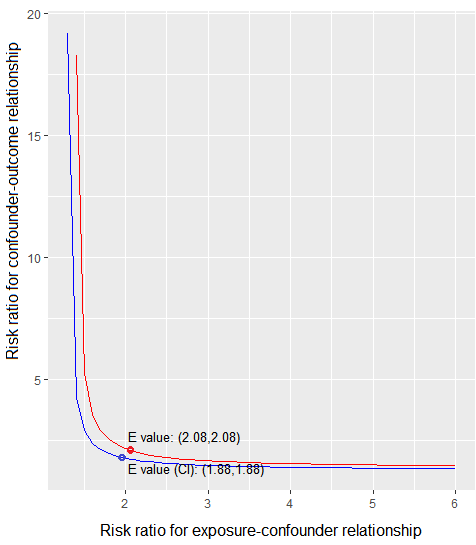
**

Figure S2. E-value demonstrating required strength of unmeasured confounder to explain observed association between PPI use and CKD risk.
